# Solidification/stabilization of lead-contaminated soil using alkali-activated volcanic ash

**DOI:** 10.1007/s11356-024-33791-z

**Published:** 2024-05-28

**Authors:** Mohammad Amin Molaei, Hania Miraki, Mohsen Morovati, Pooria Ghadir, Akbar A. Javadi

**Affiliations:** 1 School of Civil Engineering, Iran University of Science and Technology, Tehran, Iran; 2 Department of Civil and Environmental Engineering, University of Strathclyde, Glasgow, G1 1XJ UK; 3 Department of Engineering, University of Exeter, Exeter, EX4 4QF UK

**Keywords:** Alkali-activated volcanic ash, Solidification/stabilization, Lead, Soil remediation, Heavy metal, Geopolymer

## Abstract

The bioaccumulation of lead in soil poses a significant human health risk. The solidification/stabilization (S/S) technique, employing binders like Portland cement or lime, is a common method for remediating lead-contaminated soil. However, cement production has adverse environmental impacts, prompting the exploration of eco-friendly alternatives like alkali-activated materials (AAMs). This study assesses AAM efficacy in the S/S of lead-contaminated soil. The effects of several factors, including varying amounts of volcanic ash (VA), lead concentration, curing temperatures, and curing times are investigated. Unconfined compressive strength (UCS), toxicity characteristic leaching procedure test (TCLP), X-ray diffraction (XRD), Fourier-transform infrared spectroscopy (FTIR), and field emission scanning electron microscope-energy-dispersive spectroscopy-mapping analyses (FESEM/EDS/mapping) analyses are used to study the specimens. The findings indicated a substantial increase in the UCS of lead-contaminated soil treated with 15% VA (under oven curing (OC) conditions), and 10% VA (under ambient curing (AC) conditions) exhibited remarkable increases of up to 600% and 458%, respectively. Moreover, the leaching of Pb^2+^ ions from samples contaminated with 10,000 mg/kg (under OC conditions) and 2500 mg/kg (under AC conditions) experienced significant reductions of 87% (from 135.14 to 13.36 ppm) and 91% (from 26.32 to 2.21 ppm), respectively. The S/S process in these samples operated through three primary mechanisms of chemical bonding, physical encapsulation, and the formation of insoluble silicate. The formation of N-A-S–H and hydroxy sodalite structures played a vital role in facilitating these mechanisms. Therefore, alkali-activated VA demonstrated excellent performance in the remediation of lead-contaminated soil.

**Graphical Abstract:**

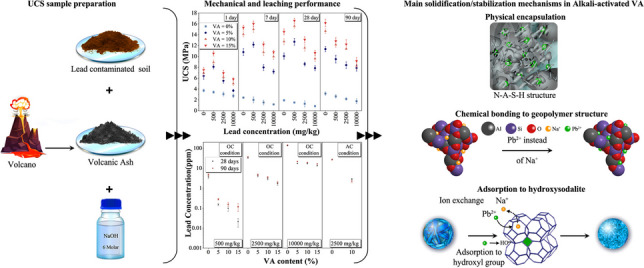

## Introduction

The bioaccumulation and toxicity of heavy metals (HMs) adversely affect human health and the surrounding environment. Various sources such as the breakdown of rocks containing heavy metals, volcanic eruptions, and human activities such as the construction of various industries, expansion of urbanization, and extraction from mines cause an increase in HMs pollution (Hamad et al. 2019; Zhuo et al. 2019; Leung et al. 2021). Lead, as a toxic heavy metal, enters the human body from two sources: dust inhalation and the food chain (Kabata-Pendias 2000). Developmental delay, learning difficulties, vomiting, weight loss, and hearing loss are some of the damages caused by lead poisoning in adults (Tang et al. 2019). Hence, developing effective technologies to remediate lead-contaminated sites is of particular importance.

Solidification/stabilization (S/S) has been broadly utilized to immobilize contaminants and improve soil characteristics. This process is implemented by introducing a binding agent into the contaminated site through which the mobility (or toxicity) of HMs is reduced, and they become encapsulated into a monolithic solid with a minor surface area (Deng et al. 2024). In the S/S process, lime (CaO or Ca(OH)2) and Portland cement are the most often utilized binders (Chen et al. 2023). However, cement production has led to multiple environmental impacts, including considerable CO_2_ emission (about 0.7–1.1 t/ t PC), dust generation, and the depletion of natural resources (Bosoaga et al. 2009).

In recent years, there has been a growing interest in a new generation of cement, called alkali-activated materials (AAM), as a substitute for Portland cement. The reaction between a reactive aluminosilicate precursor and an alkaline activator facilitates the formation of AAMs (Pacheco-Torgal et al. 2014). Specifically, when AAMs are derived from a source with a high aluminosilicate and low calcium content, they are referred to as geopolymers (Pacheco-Torgal et al. 2014). The chemical and physical properties of AAMs and geopolymers make them capable of retention of the toxic components, particularly HMs (Wang et al. 2023). The performance of AAMs in S/S is affected by numerous factors such as concentrations and types of HMs, the binder content and properties, activator content and chemical composition, curing temperature, and curing time. In soil contaminated with cadmium and lead after being treated with coal bottom ash-based geopolymer, the leaching amount of Pb and Cd decreased by 95% and 97%, respectively (Dong et al. 2023). Phair et al. (2004) showed that the immobilization of Cu^2+^ and Pb^2+^ geopolymers made with NaOH had a substantially higher immobilization efficiency than those made with Na_2_SiO_3_ or a combination of Na_2_SiO_3_ and NaOH. Investigation of different curing times and curing temperatures for fly ash–based geopolymer showed that when samples were cured at 20 °C for 28 days, the maximum level of Pb immobilization efficiency (99.99%) was attained (Nikolić et al. 2018).

When studying the efficacy of AAMs in the S/S approach, understanding the mechanisms by which AAM help in the S/S of HMs is essential. When Pb in the nitrate form was added to a metakaolin-based geopolymer, Perera et al. (2005) proposed that Pb might be encapsulated in the amorphous structure of geopolymers as no new crystalline phases were found. Lee et al. (2016a) stated Pb is dispersed in the geopolymer environment without forming a specific chemical compound. According to Phair et al. (2004), a chemical process, not physical encapsulation or sorption, is responsible for the immobilization of Pb. In addition to the mentioned mechanisms, the formation of insoluble silicate compounds such as Pb_3_SiO_5_ in AAM based on fly ash and GGBS has been mentioned in some research as a reason for Pb immobilization (Palomo and Palacios 2003; Zhang et al. 2008; Long et al. 2020).

In the last decade, the use of natural pozzolans such as volcanic ash (VA) as a precursor in AAM has been considered (Robayo-Salazar and de Gutiérrez 2018). Since there is no need for VA to be combusted before being used as a precursor and also the high amount of silicon and aluminum in VA, it is among the most suitable materials for AAM production (Takeda et al. 2014). In addition, due to the superficial nature of volcanic ash deposits and its easier extraction, the use of this material is also justified from an economic point of view. To date, the majority of research studies have primarily focused on enhancing the mechanical properties of AAMs through the utilization of VA. However, limited attention has been given to exploring the potential of AAM based on VA for S/S of heavy metals. On the other hand, there are lots of studies on the applications and mechanisms of S/S of lead using AAM mortars and pastes, but few studies have investigated the capability and mechanism of AAMs in the remediation of soil contaminated with heavy metals. Lead can pose remarkable distress to the structures through physical and chemical changes to soil texture (Li et al. 2015). Previous studies on lead-contaminated soils have shown that increasing the concentration of lead cation reduces the thickness of the diffuse double layer which leads to a reduction in the Atterberg limits, optimum moisture content, and pH and increases the maximum dry density, hydraulic conductivity, and electrical conductivity of the soil (Li et al. 2015; Nazari Heris et al. 2020). The interaction between lead-contaminated soil particles and AAMs could potentially influence the mechanisms involved in the S/S process. This interaction may yield varied outcomes compared to the S/S of lead in AMMs-based mortars and pastes, as elucidated in previous studies. Furthermore, the efficacy of alkali-activated VA in the S/S of lead contamination has received relatively scant investigation. Consequently, this study aims to capitalize on the beneficial characteristics of VA as binders in AAM and investigate their efficacy in remediating heavy metal-contaminated soil.

This study focuses on the S/S of lead-contaminated soil using alkali-activated VA. The effects of several factors, including different amounts of VA (5, 10, and 15%), different lead concentrations (500, 2500, and 10000 mg/kg^−1^), two curing temperatures (ambient cured: temperature = 25 °C, relative humidity = 85%) and oven cured (temperature = 50 °C, relative humidity = 15%), and different curing times (1, 7, 28, and 90 days) were investigated. Unconfined compressive strength (UCS) tests, toxicity characteristic leaching procedure (TCLP) tests, X-ray diffraction (XRD) analysis, Fourier-transform infrared spectroscopy (FTIR), and field emission scanning electron microscope-energy-dispersive spectroscopy-mapping analyses (FESEM/EDS/mapping) were employed to study the mechanical performance, leaching behavior, and microstructure of the samples. By investigating these factors and conducting a comprehensive analysis, this research seeks to advance our understanding of the remediation potential of AAMs based on VA, as well as the underlying mechanisms involved in the S/S process.

## Materials

**Fig. 1:**
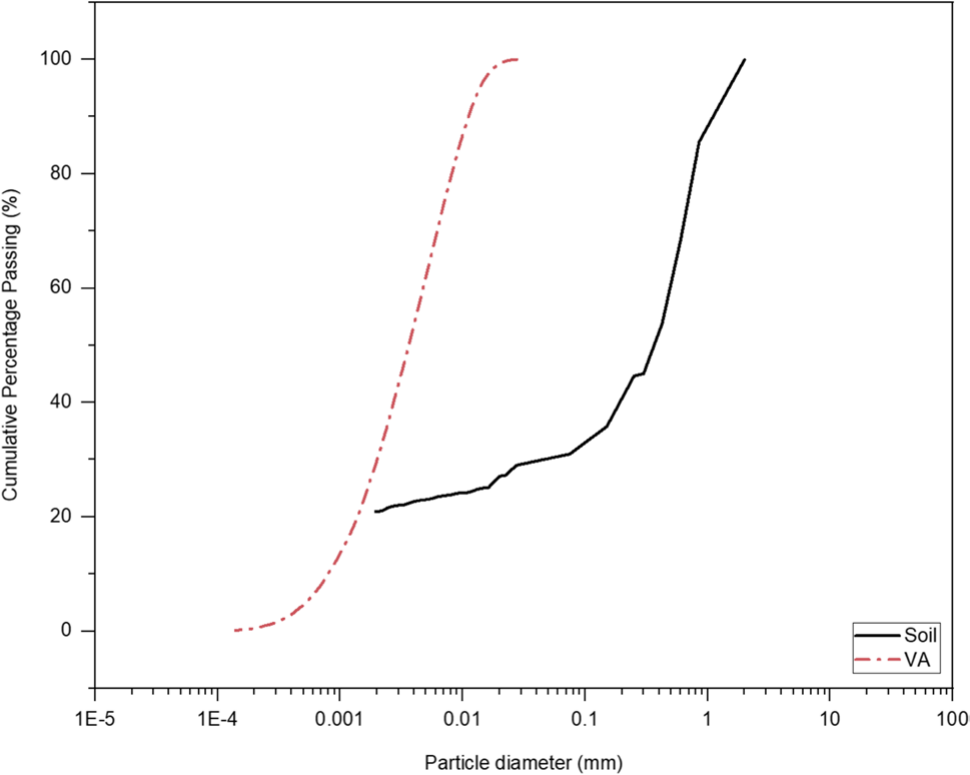
Grain size distribution of the tested soil and VA

The soil was obtained from the university campus (Tehran), and it was screened on-site through a 2-mm sieve and then dried in an oven at 105 °C for 1 day. According to ASTM D424-54 (ASTM (American Society for Testing and Materials) (1982)) and ASTM D423-66 (ASTM (American Society for Testing and Materials) (1972)), the soil’s liquid limit (LL), plastic limit (PL), and plasticity index (PI) were measured at 26%, 18%, and 9%, respectively. Particle size distribution curves were generated after sieve analysis and hydrometer testing according to ASTM D422-63 (ASTM (American Society for Testing and Materials) (2002)) and ASTM D7928-17 (ASTM (American Society for Testing and Materials((2017)), respectively (Fig. 1). The results show that the soil is classed as clayey sands (SC) soil based on ASTM 2487–11 (ASTM (American Society for Testing and Materials) (2011)). The oxide composition of the soil was investigated through X-ray fluorescence (XRF) testing, and the results are presented in Table 1. The maximum dry density (${\gamma }_{max}=1.74 \text{g}/{cm}^{3}$) and the optimum water content (${\omega }_{opt}=14\%$) of the soil were measured based on standard Proctor compaction test following the ASTM D1557-09 (ASTM (American Society for Testing and Materials) (2009)).

**Table 1:** Chemical composition of the materials used

Component oxide	Content (wt%)	
	Soil	VA
SiO_2_	49.2	55.7
Al_2_O_3_	16.8	21.8
CaO	15.3	10.1
Fe_2_O_3_	2.2	3.3
Na_2_O	1.6	5.8
MgO	2.3	1.7
K_2_O	1.9	0.9
MnO	0.8	0.1
P_2_O_5_	N.D	0.2
TiO_2_	0.9	0.3
L.O.I	9	0.1

VA utilized in this study was collected from Taftan Mountain in Iran, respectively. Grain size distributions of VA are shown in Fig. 1. The oxide compositions of the VA and soil were obtained through XRF analysis, and the results are illustrated in Table 1. Analytical grade of lead nitrate (Pb(NO_3_)_2_ provided by Merck was utilized to prepare different concentrations of contaminated soil (500, 2500, and 10000 mg/kg by the mass of the soil). To prepare the TCLP leaching solutions, acetic acid provided by Neutron Pharmachemical Company was used. The alkali activator used in this research was NaOH with a concentration of 6 M.

## Experimental program

### Overview of the tests

Two main sets of UCS tests were conducted to consider the effects of multiple factors on the S/S process. In set 1, the impacts of lead concentrations of 500, 2500, and 10000 mg/kg, various binder contents of 5, 10, and 15%, and four curing times of 1, 7, 28, and 90 days were studied, while set 2 was investigated different curing conditions (OC: cured in an oven at 50 °C with a relative humidity of 15% and AC: cured at an ambient temperature of 25 °C with a relative humidity of 85%) and four curing times of 1, 7, 28, and 90 days. The lead concentrations in set 2 were considered to be 2,500 mg/kg. The outline of the experimental program and the mix designs of the samples are shown in Tables 2, 3, and 4. Sample IDs are also shown in Tables 3 and 4. As an example, the ID V10D90-B stands for the 2500 (mg/kg) contaminated sample containing 10% VA, cured in a dry condition for 90 days. The last letter in each sample ID signifies the contamination amount in each sample, such that Un, A, B, and C stand for the 0 (uncontaminated), 500, 2500, and 10,000 mg/kg contamination in each sample.

**Table 2:** Summary of the experimental program

Set of experiments	Lead concentration (mg/kg)	Binder type	Binder replacement (%)	Activator type	Alkali activator molarity (M)	Curing time (day)	Curing condition	Activator/(soil + binder)
Set1	0, 500, 2500, 10000	VA	0, 5, 10, 15	NaOH	6	1, 7, 28, 90	Dry^a^	0.16
Set 2	0, 2500	VA	10	NaOH	6	1, 7, 28, 90	Wet^b^	0.16

^a^Temperature = 50 °C, relative humidity = 15%; ^b^temperature = 25 °C, relative humidity = 85

**Table 3:** Samples IDs of set 1 samples

Sample ID	Lead concentration (mg/kg)	Binder/soil	activator (or water)/soil
V0D^1^ (1, 7, 28, 90)^2^- Un	0	0	0.16
V5D (1, 7, 28, 90)- Un		0.05	
V10D (1, 7, 28, 90)- Un		0.1	
V15D (1, 7, 28, 90)- Un		0.15	
V0D (1, 7, 28, 90)- A	500	0	
V5D (1, 7, 28, 90)- A		0.05	
V10D (1, 7, 28, 90)- A		0.1	
V15D (1, 7, 28, 90)- A		0.15	
V0D (1, 7, 28, 90)- B	2500	0	
V5D (1, 7, 28, 90)- B		0.05	
V10D (1, 7, 28, 90)- B		0.1	
V15D (1, 7, 28, 90)- B		0.15	
V0D (1, 7, 28, 90)- C	10000	0	
V5D (1, 7, 28, 90)- C		0.05	
V10D (1, 7, 28, 90)- C		0.1	
V15D (1, 7, 28, 90)- C		0.15	

^1^D indicates the dry curing (DC) condition

^2^The numbers in the brackets indicate the curing time of each sample

**Table 4:** Samples IDs of set 2 samples

Sample number	Lead concentration (mg/ kg)	Binder/ soil	Activator (or water)/soil
V0A^1^(1, 7, 28, 90)^2^- Un	0	0.1	0.16
V10A (1, 7, 28, 90)- Un			
V0A (1, 7, 28, 90)- B	2500		
V10A (1, 7, 28, 90)- B			

^1^A indicates the ambient curing (AC) condition

^2^The numbers in the brackets indicate the curing time of each sample

### Specimen preparation

The soil was screened through a 2-mm sieve and then dried in an oven at 105 °C for 1 day. In order to prepare the contaminated soil, lead concentrations of 500, 2500, and 10,000 mg/kg were considered so lead nitrate was dissolved in deionized water (20% of the soil mass) and then added to the soil. The obtained mixture was thoroughly blended for 10 min, poured into a sealed bag, and then the mixture was allowed to stand at room temperature for 10 days to provide adequate time for chemical interactions between clay particles of soil and lead. After 10 days, the contaminated soil was dried at 105 °C for 24 h.

The activator solution content was selected as 16% of the total weight of the soil and binder. To prepare the specimens, the abovementioned contents of VA and activator solution were mixed for 5 min. Subsequently, the soil was added to the obtained slurry of both sets of the samples, and the mixing was resumed for 4 min. The mixture was then poured into standard cylindrical molds with 38 mm diameter and 76 mm height. A steel rod with a diameter of 37 mm was utilized for compaction and for eliminating air pockets. Then, the prepared specimens were demolded, and set 1 and set 2 samples were cured in OC and AC conditions, respectively, for 1, 7, 28, and 90 days. To minimize the error, for each mix design, three samples were prepared, and their average was considered the representative value.

### Unconfined compression test

Unconfined compression tests were carried out with the use of a universal testing machine with a maximum loading capacity of 50 kN and a loading speed of 1 mm/min following ASTM D2166-16 (ASTM (American Society for Testing and Materials) (2016)).

### TCLP test

The leachability of contaminated soil samples was examined by using the TCLP test (USEPA 1992). To perform this test, after subjecting the specimens to the UCS test, they were crushed to reduce the particle size to less than 9.5 mm. To prepare the leaching solution, Acetic acid was added to distilled water to reach a pH of 2.88 ± 0.05 and then mixed with crushed samples with a ratio of 20:1 for the liquid to solid, and then they were placed on a TCLP rotary machine to rotate at a speed of 30 rpm for 18 h. At the end of the test, the solution was filtered using a 0.45-μm glass fiber filter, and then 15 mL of the solution was poured into a falcon, and the lead concentration was measured using an ICP-OES device.

### Microstructural characterization

The mineral characteristics of the soil, binders, and a number of the stabilized samples were studied by XRD through an X’Pert Pro software of Panalytical Company with anode material of Cu, with a step of 0.02° over the range 2–80° operated at 40 kV and 40 mA. The PerkinElmer Company’s FTIR spectrometer was utilized to study the functional groups of the samples. Morphological analyses and element distribution were tested by field emission scanning electron microscope-energy-dispersive spectroscopy-mapping (FESEM, EDS, mapping ZEISS, Germany).

## Results and discussion

### UCS of the VA-treated soil

#### Effect of VA content

**Fig. 2:**
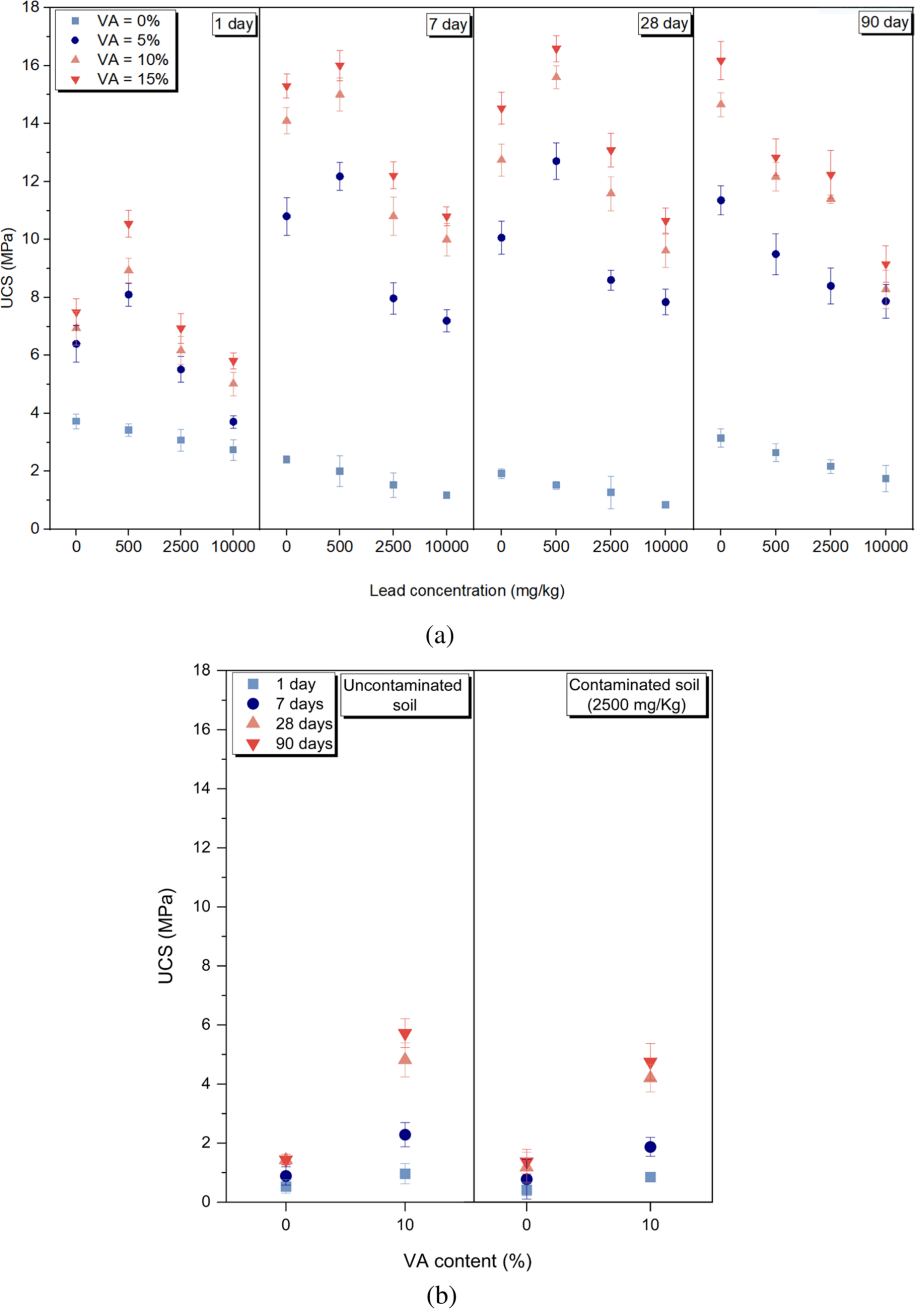
UCS of the untreated and treated samples at different curing times, **a** OC samples, **b** AC samples

**Fig. 3:**
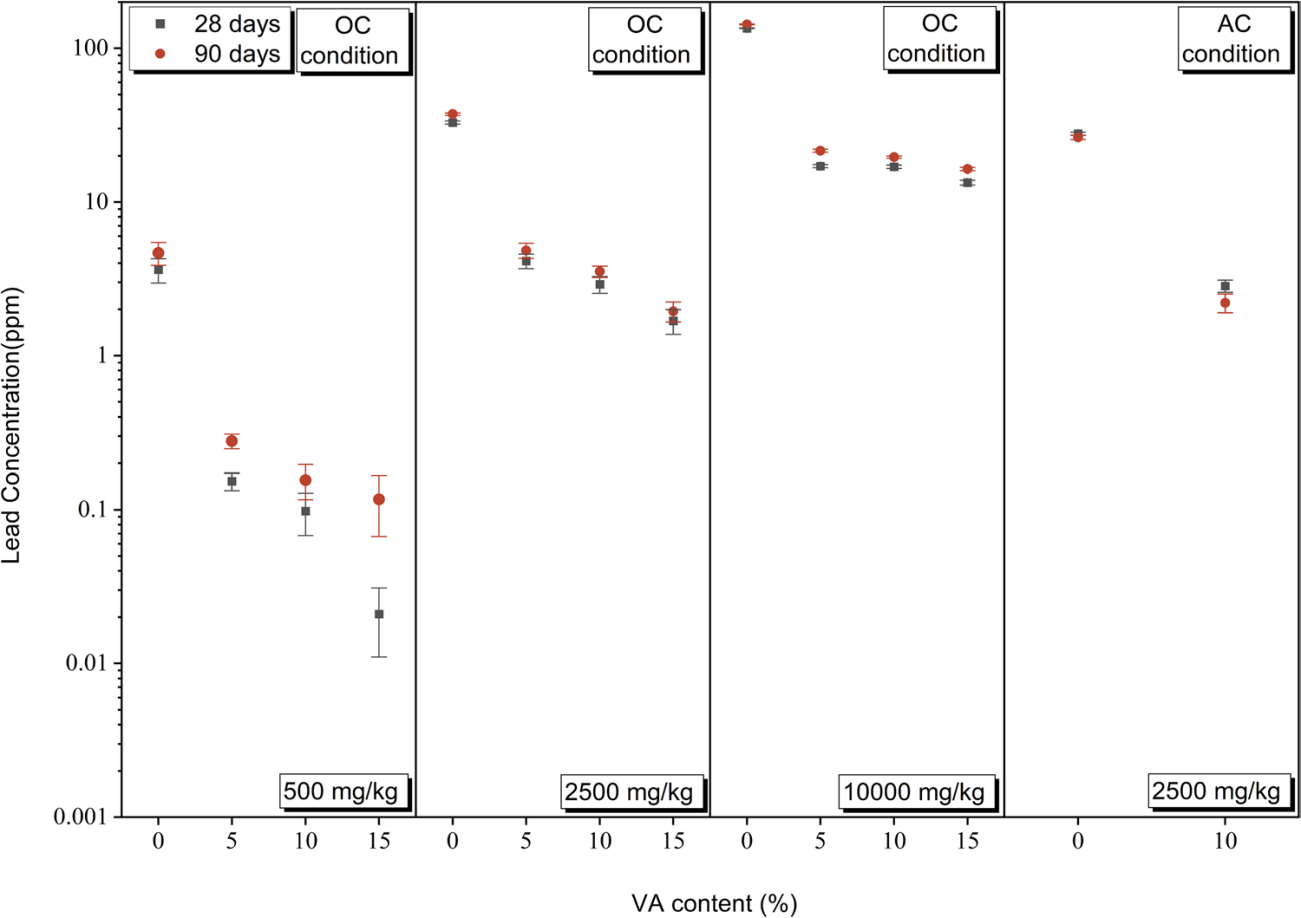
TCLP results of the untreated and treated samples

Figure 3 shows the UCS of the untreated and treated samples containing various amounts of VA and lead, at different curing times. As depicted in Fig. 2a, an increase in the percentage of VA from 5 to 15% resulted in higher UCS values, with the highest UCS achieved in samples containing 15% VA. For example, in the cases of V15D7-Un, V15D28-A, V15D28-B, and V15D28-C compared to their respective untreated samples, notable increases of 433% (from 2.91 to 15.53 MPa), 600% (from 2.51 to 17.59 MPa), 529% (from 2.27 to 14.28 MPa), and 536% (from 1.83 to 11.65 MPa) were observed, respectively. The increase in UCS at higher binder contents could be attributed to the rise in the amorphous aluminum and silicon in the medium which was dissolved by the activator leading to monomer formation (Zhou et al. 2021). The monomers’ polycondensation then led to the formation of geopolymeric networks and improved the final strength.

#### Effect of curing time and condition

According to Fig. 2a, in treated uncontaminated samples, the UCS increased up to 7 days for a constant percentage of VA. However, in the case of treated contaminated samples, the increase in UCS continued for up to 28 days. Curing conditions and moisture content have remarkable effects on the strength at various curing times. Water provides an environment and participates in the hydrolysis and dissolution of aluminosilicates, ion transfer, and polycondensation reactions. The final structure of a hardened geopolymer encompasses three distinct types of water: non-evaporable water (also known as chemically bonded water, residing within the geopolymer network), evaporable water (comprising physically bonded water derived from the geopolymerization process and integrated into the final reaction products, as well as free water occupying the larger pores of the matrix), and hydroxyl groups (found on the edges and surfaces of geopolymer colloids) (Davidovits 2008). Non-evaporable water and hydroxyl groups play pivotal roles in maintaining strength stability, with evaporation occurring within specific thermal thresholds: 100 to 300 °C for non-evaporable water and 300 to 800 °C for hydroxyl groups (Zuhua et al. 2009). Physically bonded water evaporates within the temperature range of 75 to 100 °C (Park and Pour-Ghaz 2018), while free water evaporates between room temperature and 150 °C (Perera et al. 2006). Given the curing temperatures (25 and 50 °C) applied in this study, evaporation predominantly ensues through the volatilization of free water. The higher curing temperature (in OC condition) expedited the evaporation rate of free water. The escalated evaporation rate of free water resulted in heightened alkalinity within the sample, thereby accelerating ion dissolution and polycondensation reactions. Consequently, a more compact geopolymeric network formed, leading to a pronounced enhancement of UCS. The mentioned process for treated contaminated samples occurred in 28 days. This is attributed to the presence of lead hydroxide ion which reduces the alkalinity of the medium and increases the viscosity (Lee and Van Deventer 2002). Lower alkalinity slows down the leaching of silicon and aluminum as well as polycondensation reactions. In addition, the dispersion of salt (lead nitrate) in the soil causes retardation in the geopolymerization process by reducing the evaporation rate of free pore water. This occurs through two mechanisms: (i) the increase of osmotic potential near the water evaporation levels from the soil and (ii) the formation of salt deposits on the sample surface or in the soil matrix (Nachshon et al. 2011).

In the treated uncontaminated samples, increasing the curing time from 7 to 90 days resulted in a decrease in UCS. For example, in the case of V15D7-Un compared to V15D90-Un, the UCS decreased by 17% (from 16.3 to 13.48 MPa). However, in the contaminated samples, this reduction was even more pronounced. For instance, in V15D28-C compared to V15D90-C, the UCS decreased by 34% (from 11.65 to 7.63 MPa). This could be due to two reasons. First, after 7 days, as the evaporation of free pore water increases in the soil environment, the conditions become undesirable for hydrolysis and geopolymerization, and as a result, there will be higher amounts of pores which are not filled with geopolymeric gel leading to lower UCS values (Pouhet et al. 2019) This phenomenon occurs more intensively in contaminated samples because the presence of lead in the medium of these samples and its effect in flocculating clay particles increase soil voids, and as a result, contaminated samples experience a greater drop in strength than uncontaminated samples. Second, long-term curing (90-day cured samples) can cause thermal stresses in the geopolymer matrix leading to the generation of microcracks in the internal structure of the samples which finally leads to strength reduction (Noushini and Castel 2016).

Based on the data presented in Fig. 2b, it is evident that the strength of AC samples continued to increase up to 90 days. For instance, in the case of the V10A90-B sample compared to the similar sample cured for 28 days, the strength increased by 13% (from 4.2 to 4.75 Mpa). In the AC condition, the water present in the soil medium is partially utilized in the process of geopolymerization reaction, while the rest of the water remains in the voids of the sample. Consequently, due to the low evaporation rate and continuous presence of water in the soil medium, the formation of a geopolymeric network continues for up to 90 days. Comparatively, the AC condition exhibits considerably lower UCS values than the OC condition. The geopolymerization reaction in VA-treated samples requires higher temperatures, typically ranging from 50 to 100 °C, for VA activation in the presence of an alkaline activator (Takeda et al. 2014). Moreover, higher temperatures are necessary to expedite geopolymerization reaction in alkali-activated VA samples (Fu et al. 2021). Furthermore, by reducing the evaporation rate of free pore water in the ambient-cured samples, the alkalinity of the soil environment decreases. Consequently, the geopolymerization rate decreases, leading to a reduction in the strength of AC samples when compared to OC samples.

#### Effect of lead concentration

Based on the observations from Fig. 2, it is evident that the presence of lead in all concentrations resulted in a reduction of UCS in the untreated samples. For instance, in the cases of V0D90-A, B, and C compared to V0D90-Un, the UCS decreased by 16, 31, and 44%, respectively. Lead reduces the thickness of the diffuse double layer of the clay minerals. This finally results in the agglomeration of clay particles and the generation of more empty spaces among soil particles resulting in a porous structure (Li et al. 2015; Nazari Heris et al. 2020), which can reduce the UCS of the contaminated soil.

In the case of treated contaminated samples, with the constant VA content and specific curing time, the introduction of the lead up to 500 mg/Kg (group A) had a positive effect on the UCS. For example, in V5D1-A, V5D7-A, and V5D28-A samples, the UCS respectively increased by 26.5% (from 6.4 to 8.1 MPa), 11.6% (from 11.80 to 13.17), and 24% (from 11.06 to 13.71 MPa) compared to the similar uncontaminated samples. Increasing the concentration of lead up to 2,500 (group B) and 10,000 (group C) mg/kg reduced the UCS. The V5D28-B and V5D28-C samples experienced 13% (from 11.06 to 9.6 MPa) and 20% (11.06 to 8.84 MPa) reduction in strength, respectively compared to the similar uncontaminated sample.

It seems that increasing lead up to a certain concentration (group A samples in this research) can modify the geopolymerization process and the formation of geopolymeric network (Zhang et al. 2008; Pu et al. 2021). With the increase in lead concentration (groups B and C), the capacity of the geopolymeric network to use lead as a modifier becomes exhausted (Yunsheng et al. 2007), and lead caused adverse effects in the geopolymerization process, and as a result, the formation of the geopolymer structure was reduced or limited. The adverse effect of lead nitrate on the compressive strength of geopolymers has already been reported in various studies. Adding 1% of lead nitrate to fly ash–based geopolymer reduced the compressive strength by 45% compared to the uncontaminated sample (Lee et al. 2016b). Adding 1% of lead nitrate to geopolymer based on Shell coal gasification fly ash reduced the compressive strength by 30% (Chen et al. 2022). The adverse effect of lead occurs by the substitution of lead cations with sodium or potassium cations in the three-dimensional structure of geopolymer to neutralize the negative charge of the final product (Ogundiran et al. 2013; Muhammad et al. 2018) which might affect the integrity and uniformity of the geopolymer structure. Excess lead can disrupt the poly-condensation process and make it difficult for the components to link together during the geopolymerization process. Hence, the expansion of the geopolymeric chain remains incomplete leading to a reduction in UCS (Nikolić et al. 2018; Wang et al. 2018). Nevertheless, the ultimate strength values of all of the treated samples were remarkably higher than those of contaminated-untreated soil and could successfully meet the minimum strength of 2.068 MPa as stated by Portland Cement Association (Portland Cement Association 1971) for cement-stabilized base and subbase.

Based on Fig. 2, it can be observed that at a curing time of 90 days and a constant VA content, an increase in lead concentration led to a decrease in UCS across all concentrations. The highest decrease in UCS was observed between V15D90-Un and V15D90-C samples, where the strength decreased by 76% (from 13.48 to 7.63 MPa). When the samples were cured under dry conditions for up to 90 days, the presence of lead ions resulted in larger pores left behind due to the evaporation of free water. Consequently, this led to a further decline in UCS. Moreover, in B and C groups, due to the higher concentration of lead compared to group A, the mechanism of reduction of DDL thickness and formation of flocculated structure occurs more intensively, and as a result, more and larger pores are formed and the reduction in UCS will be greater.

### TCLP results

Figure 3 shows the TCLP results of samples containing different concentrations of Pb^2+^. As shown in Fig. 3, for a fixed lead concentration, an increase in binder content led to a decrease in lead leaching. In the case of group A samples, the reduction in leaching for V15D28-A and V15D90-A was 99.5% (from 3.62 to 0.02 ppm) and 97.5% (from 4.66 to 0.11 ppm), respectively. Similarly, for group B samples, the reduction values were 94.9% (from 32.87 to 1.64 ppm) and 94.8% (37.32 to 1.94 ppm), while for group C samples, the reductions were 88.5% (from 143.21 to 16.41 ppm) and 90% (from 135.14 to 13.36). Comparing the TCLP results of group A and group B samples with the EPA standard reveals that all treated samples had a leaching concentration below 5 ppm, thus categorizing them as non-hazardous materials (USEPA 1992).

The formation of a geopolymer network can lead to physical encapsulation through which heavy metals are trapped within the oligomeric coating gels (Guo et al. 2017; Wang et al. 2018), leading to leaching prevention. In the samples treated with a fixed amount of VA, Increasing the curing time from 28 to 90 days led to an increase in lead leaching in all samples. Cracks caused by thermal stresses can disrupt the performance of the physical encapsulation mechanism, when these cracks are exposed to TCLP liquid and lead particles are washed and leached from the surfaces of these cracks. Despite the significant decrease in the UCS with increasing the curing time from 28 to 90 days, there was a very slight increase in lead leaching in VA-treated samples. This could be due to the chemical bonding of lead in the geopolymer network. This chemical bond occurs through the exchange of metal cations with Na^+^ and bonding as a result of the interaction between the anions and metal ions (Ji and Pei 2019a). For a consistent VA content and curing time, it was observed that the leaching of Pb increased proportionally with higher initial Pb concentrations. Despite the synergic occurrence of both physical encapsulation and chemical bonding, the leaching in the highest concentration (group C samples) failed to be dropped to the required level recommended by the EPA standard. This could be attributed to the high concentrations of lead that disrupted the development of geopolymeric chain and made the occurrences of physical encapsulation difficult. Also, the capacity for the chemical bonding between lead cations and the geopolymer network is augmented at lower lead concentrations; hence, the excess lead leaches in the soil medium.

In the comparative evaluation of curing conditions for group B samples treated with 10% VA, it was observed that although there was a notable contrast in UCS outcomes, lead leaching exhibited a similar reduction level under both curing conditions. This suggests that, under AC conditions, the formation of the geopolymeric network was adequate to mitigate lead leaching through mechanisms involving physical encapsulation and chemical bonding. Consequently, the excessive formation of geopolymeric structures and the considerable enhancement in UCS under OC conditions did not significantly contribute to further reducing lead leachability.

### Microstructural analysis

#### XRD results

**Fig. 4:**
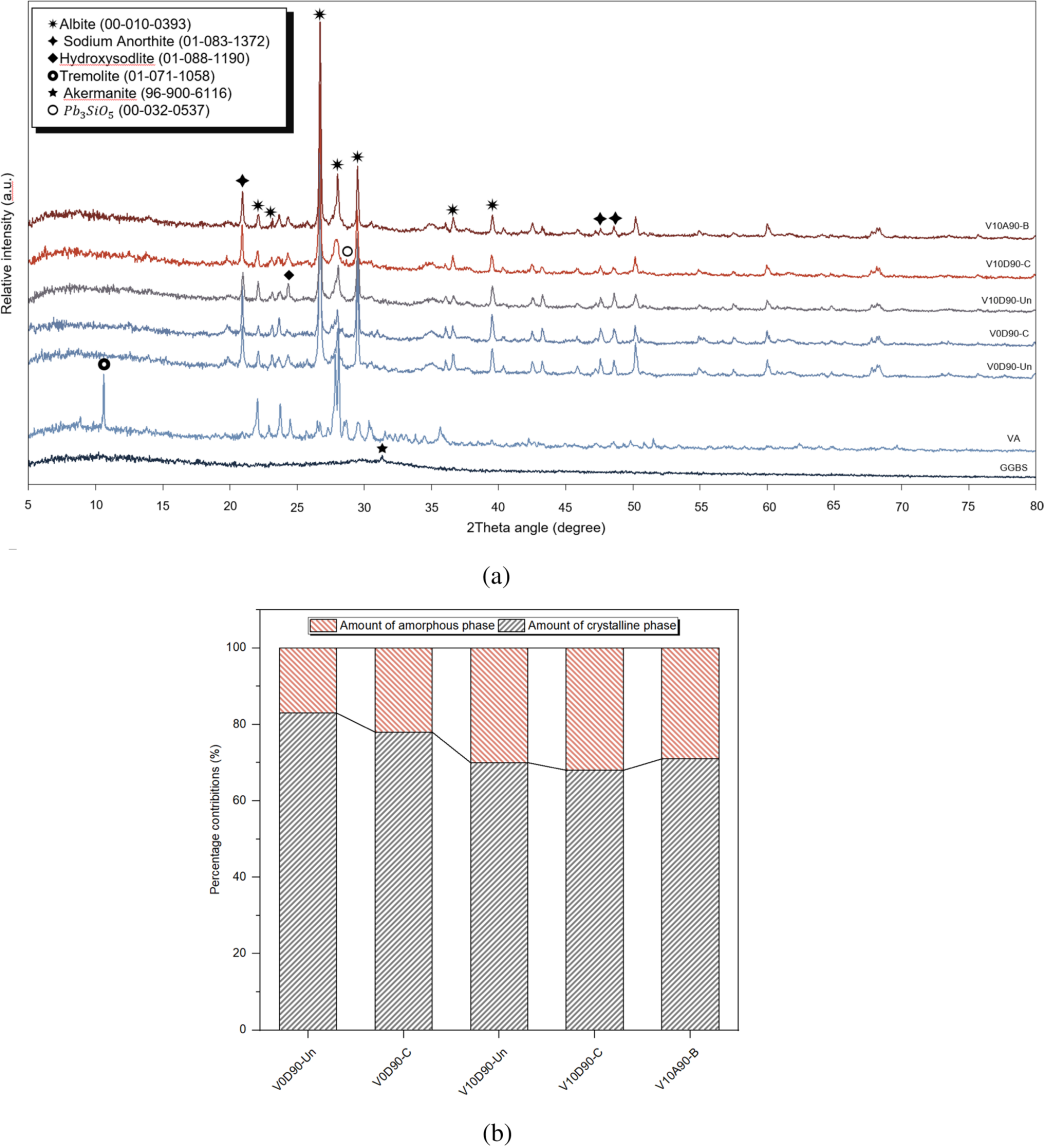
XRD patterns of the binders, untreated soil, and treated soil (**a**), and crystalline and amorphous phases of untreated soil and treated soil (**b**)

Figure 4a shows the XRD patterns of V0D90-Un, V0D90-C, V10D90-Un, V10D90-C, and V10A90-B samples. Using Origin Pro software, a quantitative analysis of the XRD data was performed in order to determine the amount of amorphous phase. The percentages of amorphous and crystalline phases for all samples are presented in Fig. 4b.

In Fig. 4a, the XRD pattern of VA exhibited several sharp peaks which are attributed to sodium anorthite (PDF 96–900-6116), tremolite (PDF 01–071-1058), and albite (PDF 00–010-0393). In the treated samples, the intensity of albite at 2*θ* of 23.6°, 21°, 26°, and 36.6° and anorthite peaks at 2*θ* angles of 20.1° and 47.6° decreased, and the tremolite crystalline phase of VA was completely destroyed which was due to the dissolution of silicon and calcium in the medium and provided the conditions for the formation of geopolymeric reaction products.

Determining the amount of amorphous and crystalline phases of each sample is beneficial for better understanding the phenomenon of geopolymerization. In Fig. 4b, the increase in the amorphous phase of samples V10D90-Un, V10D90-C, and V10A90-B indicates the alkaline activation of binders and the development of geopolymer network. A slight increase in the amount of amorphous phases in samples cured in dry conditions compared to ambient conditions shows the ability of oven curing in the dissolution of aluminosilicates and the formation of geopolymeric amorphous phases. The full width at half maximum (FWHM) depends on the crystallite size such that its lower amounts indicate higher crystallinity and more ordered structure. In Fig. 4a, the FWHM value for the albite phase at the 2*θ* of 29.49° in the V0D90-Un and V0D90-C samples were 0.18 and 0.15, respectively, and for the V10D90-Un and V10D90-C samples, the FWHM values were obtained as 0.38 and 0.40, respectively. This increase in the value of FWHM in the treated samples can indicate the formation of amorphous geopolymeric phases such as N-A-S–H in these samples.

In Fig. 4a, the increase in the intensity of the peak in V10D90-Un and V10D90-C samples at an angle of 24.3 degrees is related to the formation of hydroxy sodalite which is a type of zeolite (01–088-1190). Heating can rapidly change the aluminosilicate nature. The more the heating progresses, the sharper the peaks become which are identified as zeolite-type structures (Fletcher et al. 2005; Fultz and Howe 2012). In the V10D90-C sample, small peaks were observed at the angles of 27.7° and 28.7°, which could be related to the reaction of lead with dissolved silica and the formation of Pb_3_SiO_5_ (PDF 00–032-0537). This indicates another type of lead interference in the geopolymerization process. The formation of such insoluble compounds can contribute to reducing the leaching of lead from treated samples. The formation of such compounds with low solubility has been reported in previous studies in samples with higher lead concentrations (3.125% or 2%) (Palomo and Palacios 2003; Palacios and Palomo 2004; Zhang et al. 2008; Long et al. 2020). In general, the formation of Pb_3_SiO_5_ is highly probable in the medium with high lead content; however, in this research, the highest concentration of utilized lead was 1%.

#### FESEM/EDX analysis

**Fig. 5:**
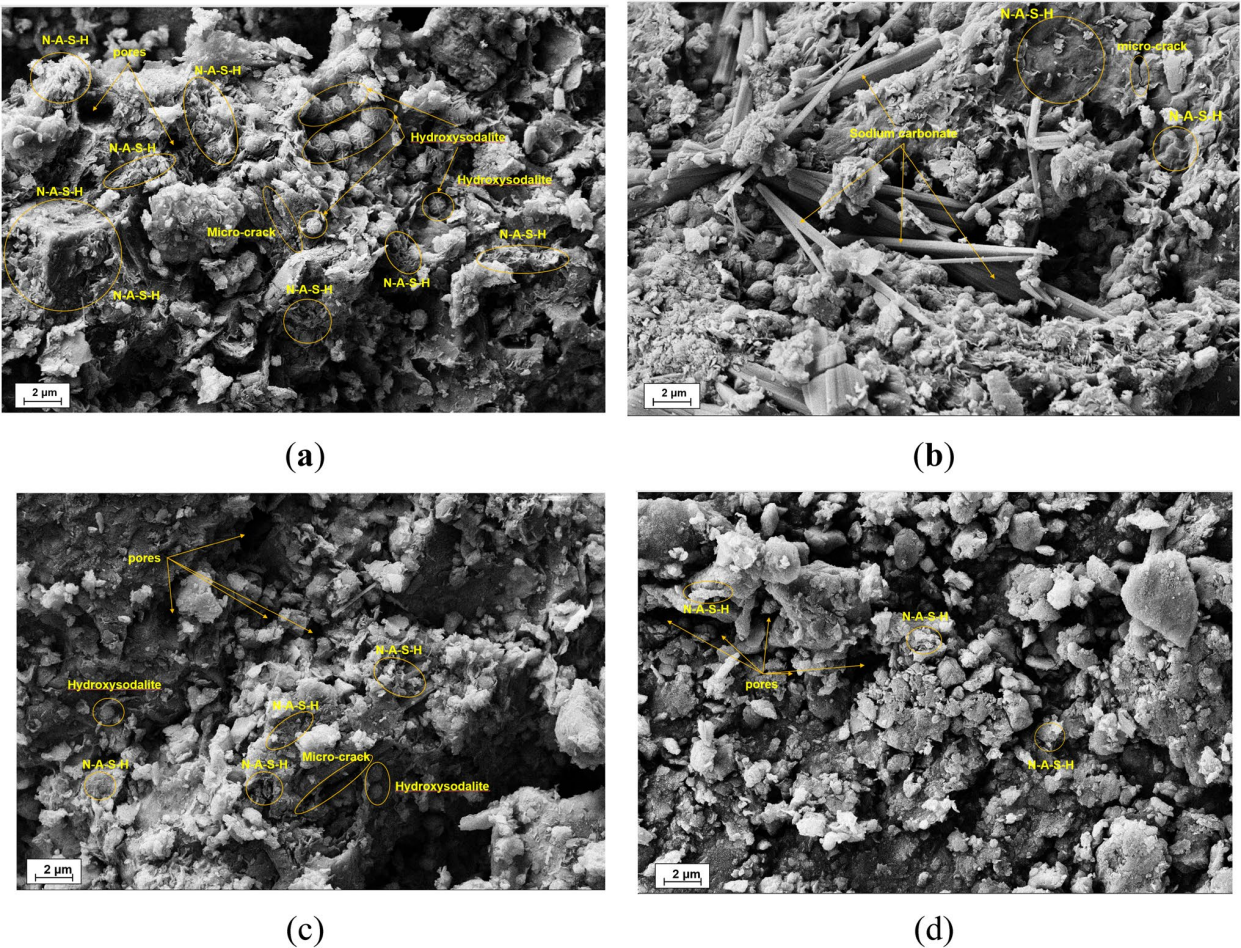
FESEM micrographs of **a**, **b** V10D90-Un, **c** V10D90-C, **d** V10A90-B samples

Figure 5 indicates the FESEM micrographs of the V0D90-Un, V0D90-C, V10D90-Un, V10D90-C, and V10A90-B samples. In Fig. 5a, the N-A-S–H gel and the crystal structure of wool ball–like particles of hydroxy sodalite are observed. The possibility of the formation of these compounds was discussed in the XRD section. The formation of zeolite crystal structures such as hydroxy sodalite along with the N-A-S–H structure can help to improve the compressive strength of geopolymers (Lee et al. 2016a), which was confirmed by the UCS results of this research.

Some needle-shaped crystals are also observed in Fig. 5b. These crystals can be formed through the reaction of sodium which has not participated in geopolymeric processes with carbon dioxide of the surrounding environment (Shilar et al. 2022). In Fig. 5c, formed gels are more dispersed and less dense compared with the V10D90-Un sample. This could be tied to the adverse effect of lead ions on the formation of the geopolymeric network. A number of microcracks are observed in Fig. 5a, b, and c. These microcracks are mainly generated due to thermal stresses caused by the long-term curing of geopolymers in dry conditions. In addition to these microcracks, some pores are also observed in the structure of different samples. These pores are formed due to the evaporation of water during the geopolymerization reaction and long-term oven curing conditions.

**Fig. 6:**
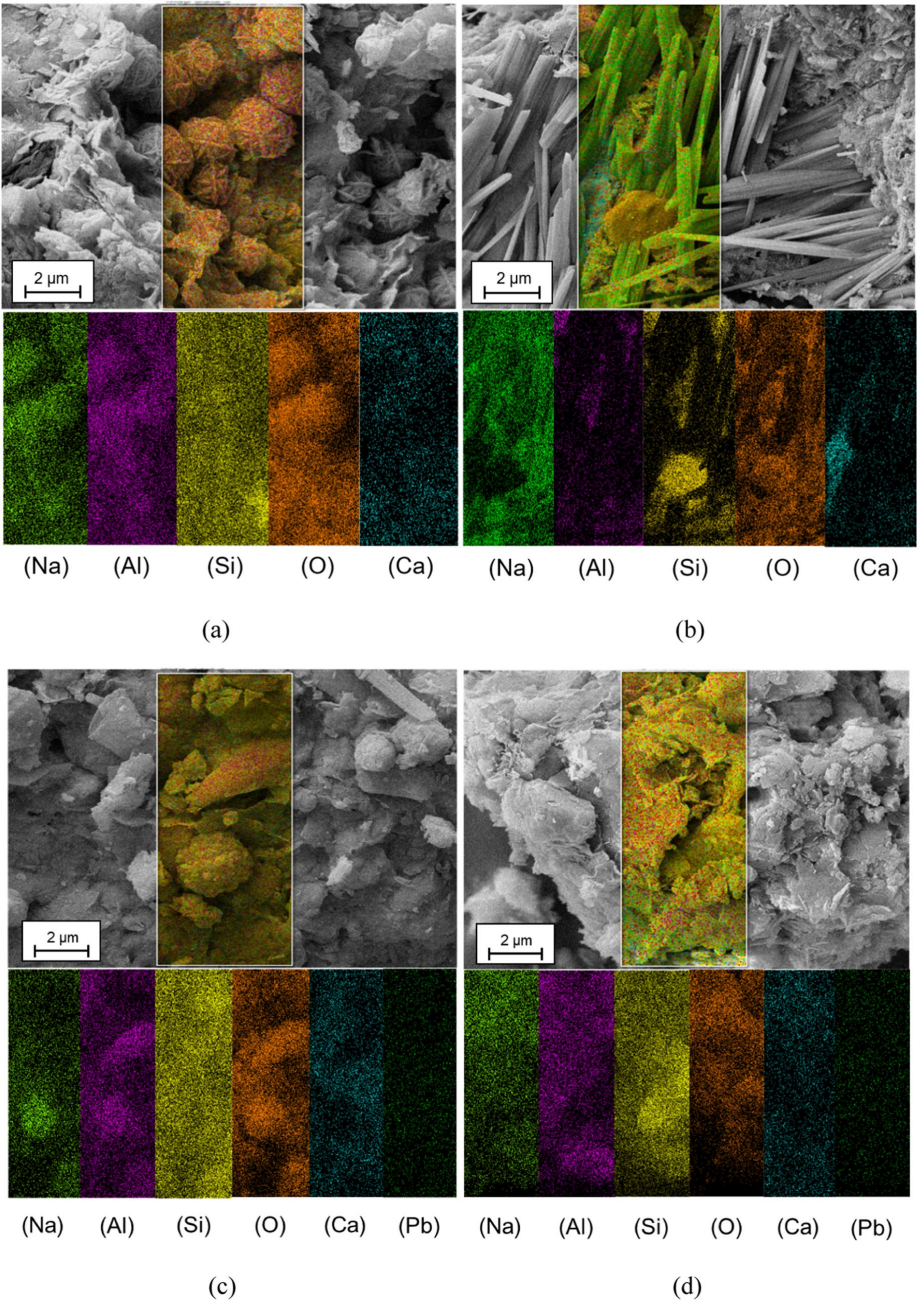
Mapping analysis of **a**, **b**V10D90-Un, **c** V10D90-C, **d** V10A90-B

Figure 6 shows the mapping analysis of V10D90-Un, V10D90-C, and V10A90-B samples. The uniform dispersion of lead ions contributes to better bonding among formed compounds and provides the appropriate conditions for lead participation in geopolymeric reactions (Tan et al. 2019). The mapping analysis of V10D90-Un and V10D90-C samples indicates the presence of sodium, silicon, aluminum, and oxygen, which are the main compounds of hydroxy sodalite, confirming its formation in the matrix. In addition, this proper distribution of the mentioned elements provides the conditions for the formation of N-A-S–H gel.

The mapping analysis of the V10D90-C sample indicates some changes in the texture and structure of the hydroxy sodalite. The adsorption of lead in the structure of hydroxy sodalite as a zeolite structure (Golbad et al. 2017; Fan et al. 2021) is one of the reasons for leaching prevention and insolubility of lead in the treated samples. Adsorption of Pb^2+^ to the structure of hydroxysodalite can be done through adsorption to the hydroxyl group and ion exchange with sodium (Lv et al. 2022). Also, the study conducted by Lee et al. (2016b) demonstrated that increasing the concentration of lead hindered the formation of hydroxy sodalite in fly ash–based geopolymer. Figure 6b is related to the mapping of the needle-shaped crystal structure of sodium carbonate.

**Fig. 7:**
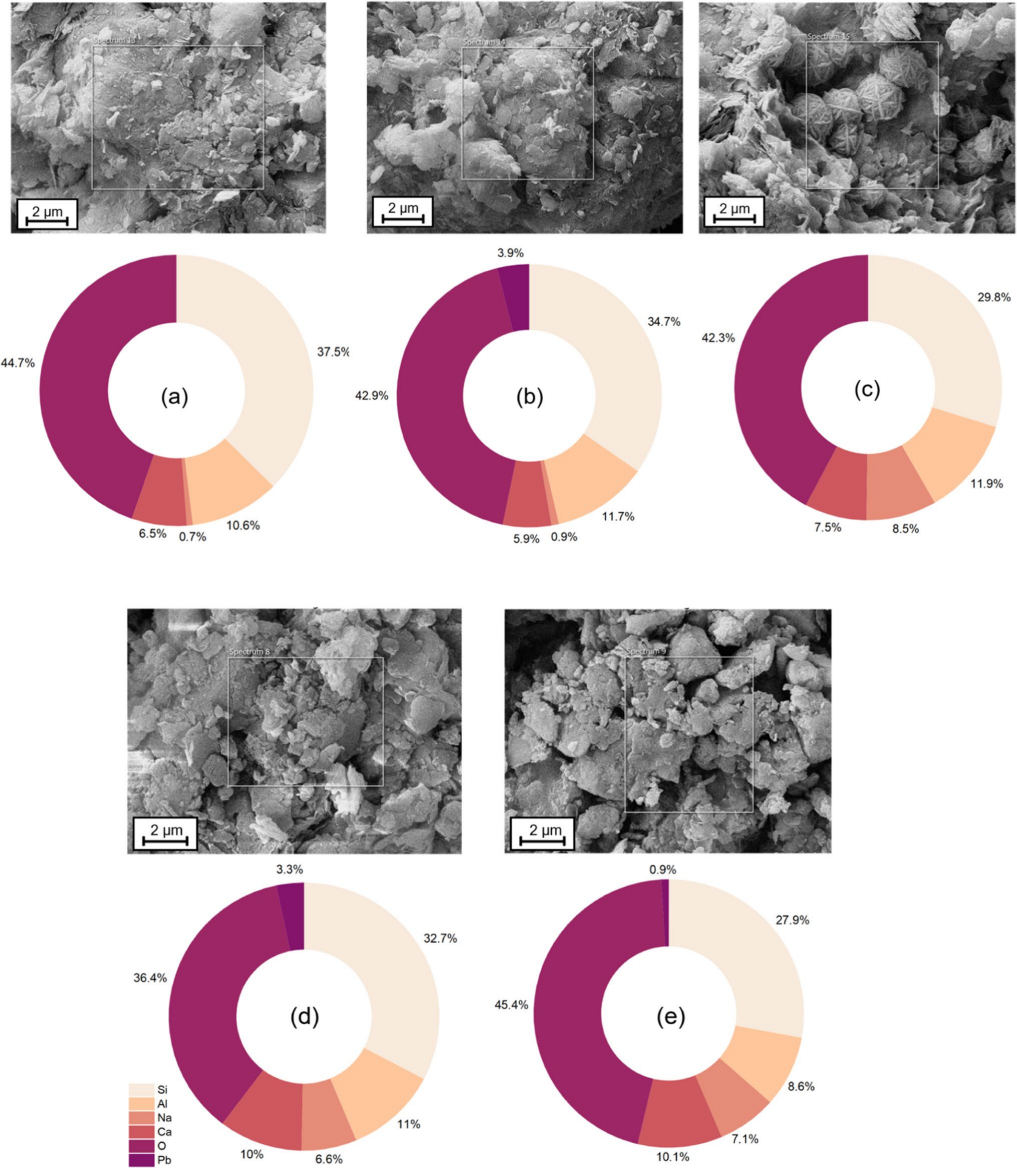
EDX analysis of **a**V0D90-Un, **b**V0D90-C, **c** V10D90-Un, **d** V10D90-C, **e** V10A90-B

Figure 7 indicates the EDS analysis of V0D90-Un, V0D90-C, V10D90-Un, V10D90-C, and V10A90-B samples. The sodium content in V0D90-Un and V0D90-C samples is very low which is due to the absence of geopolymer in these samples. Higher sodium contents were detected in V10D90-Un, V10D90-C, andV10A90-B samples which, along with the proper presence of silicon, aluminum, and calcium ions, provided the suitable conditions for the formation of N-A-S–H gels (Bilondi et al. 2018). Additionally, Si/Al and Na/Al ratios are elemental ratios for geopolymer formation (Chen et al. 2018) which are within the appropriate range for all treated samples. As observed, the atomic ratio of Si/Al increased in sample V10D90-C compared to sample V10D90-Un. This could be due to the increase of Pb concentration (on a nano-structural level) in the matrix. Hence, it could be perceived that either amorphous “lead-incorporated aluminum-deficient” aluminosilicate gel or amorphous lead silicate has been formed as a result of Pb contamination (Nikolić et al. 2018). This is in agreement with XRD analysis and identification of the Pb_3_SiO_5_ phase in the V10D90-C sample.

#### FTIR analysis

**Fig. 8:**
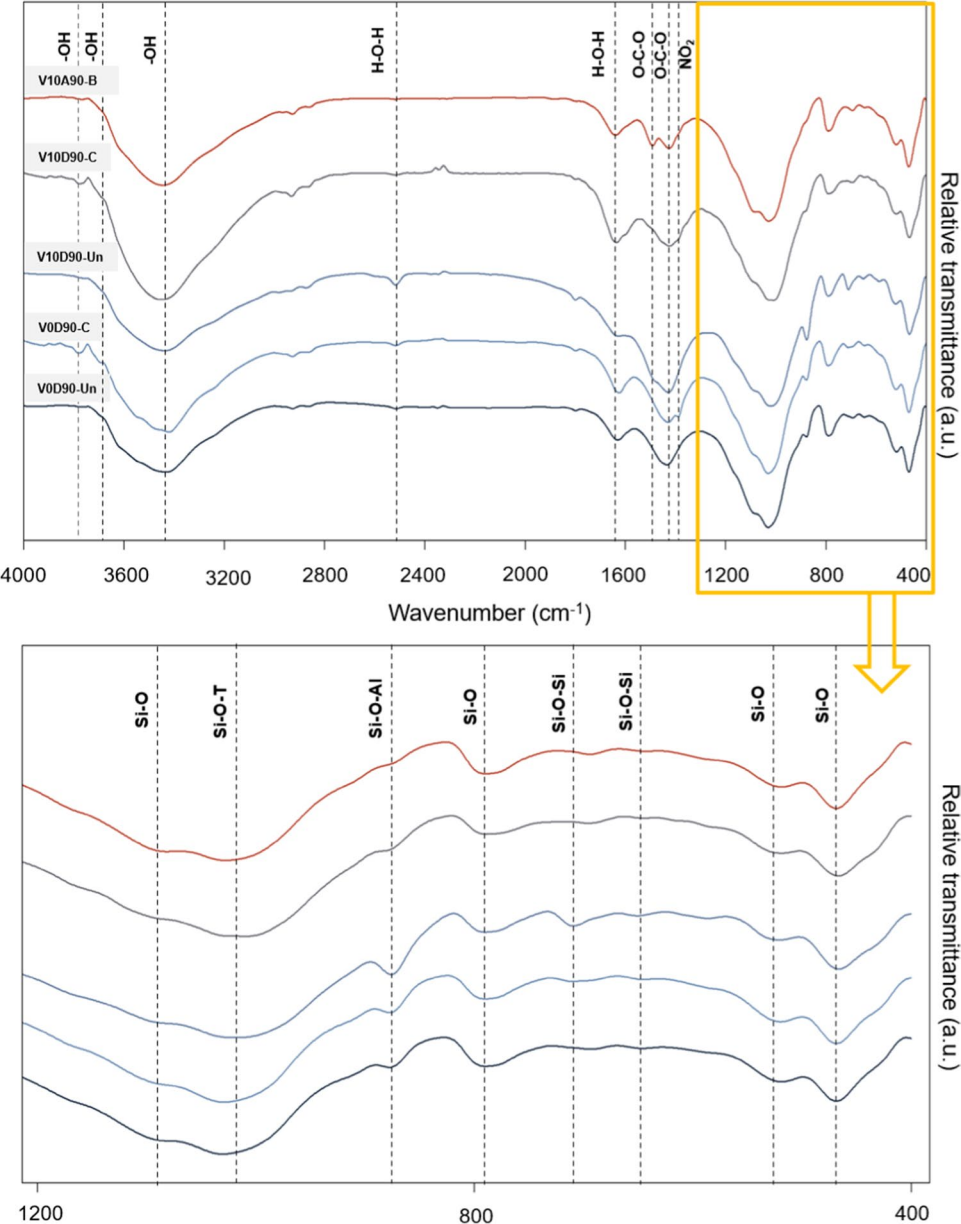
FTIR spectra of untreated and treated samples

FTIR spectra of V0D90-Un, V0D90-C, V10D90-Un, V10D90-C, and V10A90-B samples are shown in Fig. 8. Some bands at wave numbers of about 460, 520, and 1080 cm^−1^ were vivid in all the samples which are due to Si–O stretching and bending vibrations and are associated with the main components of the soil structure. The OH^−^ absorption band at wave numbers 3420 cm^−1^ to 3452 cm^−1^ and 3770 cm^−1^ and the H–O-H absorption band at wave numbers 1623 to 1637 cm^−1^, 1794 cm^−1^, and 2520 cm^−1^ correspond to crystalline water and physical water respectively. Absorption of moisture from the atmosphere as well as water entrapment in geopolymer cavities can cause the formation of physical water (Fernández-Jiménez and Palomo 2005). The process of geopolymerization is done properly in the presence of water and heat; hence, during the geopolymerization process, more water is drawn into the soil structure and geopolymer, which results in increased formation of hydroxyl bonds (Fernández-Jiménez et al. 2008). The absorption bands in the wave numbers 1407 to 1489 cm^−1^ and the two weak bands in 2861 and 2828 are related to the O-C-O bond which is due to the carbonation in geopolymers and the formation of structures such as sodium carbonate (Mijarsh et al. 2014). The absorption band (NO_2_) that occurred in the wave number 1385 is attributed to the nitrate bond owing to the soil contamination with lead nitrate (Ji and Pei 2019b).

The bands appearing around the wave numbers 646 cm^−1^, 587 cm^−1^, 870 cm^−1^, and 1000 cm^−1^ correspond to the flexural vibrations of Si–O bonds in the tetrahedral SiO_4_, the symmetrical vibrations of Si–O-Si bonds, and tensile vibrations of Si–O-Al bonds (Luo et al. 2022). These bands are the main bands in the aluminosilicate matrix of a geopolymer gel which are known as the fingerprint of geopolymerization area (Palomo et al. 1999; Yaseri et al. 2019). These absorption bands are susceptible to the presence of Al and Si. The replacement of AlO_4_ tetrahedral structures with SiO_4_ as a result of the expansion of geopolymerization reactions and the creation of the interconnected geopolymer network can be explained by the fact that Al-O bonds are weaker than Si–O bonds, which is why these bands have been displaced to lower wave number (Davidovits 2008). The shift and reduction of the wave number (from 1030 to 1008 cm^−1^) of the Si–O-T band in treated samples compared to untreated samples implies the formation of geopolymeric matrix. On the other hand, FWHM values for this band in V0D90-Un and V0D90-C samples were 196 and 188 cm^−1^, respectively; while in the treated samples, the value of FWHM was in the range of 202 to 228 cm^−1^. Such increase in the value of FWHM confirms an increase in the amount of amorphous compounds and the formation of more geopolymeric structures such as N-A-S–H in the treated samples (Rees et al. 2007a, ul Haq et al. 2014). Furthermore, the increase in the intensity of the Si–O-Si band in the V10D90-Un sample at wave numbers of 870, 587, and 646 cm^−1^ can be attributed to the further development of the geopolymeric network (Rees et al. 2007a; 2007b).

In the V10D90-C sample, the lead metal reduced the Si–O-T bond wave number to 1008 cm^−1^ which is the lowest wave number in this bond. This indicates the involvement of lead in the formation of geopolymer structures (Ji and Pei 2019a; Xia et al. 2019). Studies have shown that the position of the Si–O-T band has changed in the presence of heavy metals such as zinc and lead, which could be due to the presence of these heavy metals as substitutions for charge-balancing ions (sodium and calcium) in the geopolymer structure (Xia et al. 2019). This also confirms the occurrence of physical encapsulation of lead in the geopolymer structure (Muhammad et al. 2018). Symmetric resistive vibration at wave number 790 cm^−1^ corresponds to the Si–O-Si bond which is related to inactivated geopolymeric raw materials. But considering that this band is also present in the structure of the used soil, it can be investigated through a change in its intensity which is related to inactivated geopolymeric raw materials (Yaseri et al. 2019). The dissolution of Si and its separation from this bond to participate in the geopolymerization process reduces the intensity of this bond in V10D90-Un and V10D90-C samples (Hajimohammadi et al. 2017).

The absorption band at wave number 710 cm^−1^ is related to the formation of hydroxy sodalite in samples V10D90-Un and V10D90-C (Klima et al. 2022). The reduction of this wave number to 690 cm^−1^ in sample V10D90-C could indicate the intervention of lead in the formation of Si–O-Al and/or hydroxy sodalite bonds. This was also seen in the FESEM-mapping analysis.

## Discussion

During the geopolymerization process in samples treated with alkali-activated VA, the formation of N-A-S–H structures, accompanied by the emergence of hydroxy sodalite (HS) zeolite structures and the detection of the Pb_3_SiO_5_ structure (in group C), was observed. This was evidenced through XRD, FTIR, and FESEM-EDX-mapping analyses. Previous studies (Djobo et al. 2017) have also noted the formation of N-A-S–H and zeolite structures (zeolite Y and HS) in alkali-activated VA. The simultaneous formation of N-A-S–H structures and the uniform distribution of lead particles within the soil matrix, as observed in FESEM-mapping images, create favorable conditions for lead stabilization within these structures via physical encapsulation and chemical bonding mechanisms. Moreover, HS exhibits notable adsorption properties for heavy metals (Esaifan et al. 2019), with its formation previously documented in fly ash (Sukmak et al. 2013), coal bottom ash (Geetha and Ramamurthy 2013), metakaolin (Granizo et al. 2014), and volcanic pozzolan–based geopolymers (Moon et al. 2014). The likelihood of HS formation increases with activators possessing lower silica content and higher alkalinity (Rożek et al. 2019). Reported Si/Al ratios ranging from 0.5 to 2.46 (Fletcher et al. 2005; Khan et al. 2015; Król et al. 2016), sodium hydroxide activator molarities of 5, 8, and 12 (Álvarez-Ayuso et al. 2008; Moon et al. 2014; Bobirică et al. 2015), and curing conditions of 1 day at 60 °C or 7, 28, and 180 days at 85 °C (Criado et al. 2010; Ozer and Soyer-Uzun 2015) have been linked to HS formation. In our study, the curing conditions (1, 7, 28, and 90 days at 50 °C), a Si/Al ratio of 2.55, and the utilization of an 8 M sodium hydroxide activator appear to have facilitated HS formation. This phenomenon contributed to enhanced mechanical strength and reduced lead leaching.

In this investigation, all three primary mechanisms of S/S of heavy metals in geopolymers—namely, physical encapsulation, chemical bonding, and the formation of new chemical structures of heavy metals (Ji and Pei 2019b)—contributed to the remediation of lead-contaminated soil with chemical bonding proving to be the most effective mechanism. The validation of the physical encapsulation mechanism was achieved through the correlation of results from UCS, TCLP, XRD, FTIR, and FESEM-EDX-mapping analyses. Confirmation of the chemical bonding mechanism was obtained through TCLP, FTIR, XRD, and FESEM-EDX-mapping analyses, while validation of the formation of a new chemical structure mechanism (Pb_3_SiO_5_ formation) was achieved via XRD and FTIR analyses. Previous studies on the S/S of lead using metakaolin- and kaolinite-based geopolymers have reported mechanisms involving physical encapsulation and the formation of silica- and carbonate-based structures (El-Eswed et al. 2015; El-Eswed et al. 2017). In fly ash–based geopolymer, physical encapsulation (Van Jaarsveld et al. 1998, Van Jaarsveld and Van Deventer 1999, Phair et al. 2004, Nikolić et al. 2014, Lee et al. 2016b, Nikolić et al. 2018, Wang et al. 2018), chemical bonding (Van Jaarsveld et al. 1998, Van Jaarsveld and Van Deventer 1999; Nikolić et al. 2014; Wang et al. 2018), surface adsorption (El-Eswed et al. 2015; Wang et al. 2018), and formation of new silica- and carbonate-based phase (Palomo and Palacios 2003, Palacios and Palomo 2004, Phair et al. 2004, Zhang et al. 2008, Guo et al. 2017) mechanisms were reported. The observed variations in mechanisms can largely be ascribed to several factors, including the physicochemical properties of the binder, the type of matrix utilized for the (S/S) process (mortar, paste, and soil), the type of activator (sodium hydroxide, sodium hydroxide/sodium silicate, and potassium hydroxide), the curing temperature (ranging from 25 to 95 °C), and the type and concentration of lead species (PbSO_4_, PbO, PbS, Pb(NO_3_)_2_). These parameters can influence the interactions between lead cations and geopolymerization reactions, potentially impacting the mechanisms involved in the S/S of lead using AAMs. This study stands out from prior research in this domain by utilizing VA as a binder and soil as the base matrix in the S/S process with AAMs. The uniform dispersion of lead within the soil structure (attributed to a 10-day pre-experiment contamination period), the interplay of lead-contaminated soil particles with geopolymerization reactions, and the favorable chemical reactivity of VA are identified as the primary factors contributing to the concurrent occurrence of all three mechanisms.

Increasing the binder content typically fosters more favorable conditions for the formation of geopolymerization products, thus augmenting the S/S process. However, in this investigation, the correlation between the rise in UCS and the decline in lead leaching did not demonstrate a linear trend with escalating binder content. The most substantial alterations were observed when the binder content increased from 0 to 5%. Conversely, with higher VA amounts (10 and 15%), the variations in strength and leaching content were less pronounced. This discrepancy could be attributed to several factors, including the insufficient quantity of activator to activate higher amounts of VA fully, inadequate curing temperature to complete all geopolymerization reactions, and the conversion of free sodium ions to sodium carbonate phase during carbonation without contributing to the N-A-S–H formation. Consequently, undissolved and unreacted particles were likely present in the soil medium. Their non-participation in geopolymerization reactions led to imperfections in the geopolymeric network, thereby reducing the rate of strength gain with increasing binder content. This hypothesis supports the observation of unreacted particles and the formation of sodium carbonate structures in the treated samples, as revealed by FTIR and FESEM-EDX-map analyses in previous sections.

Under OC conditions, a higher UCS was attained compared to AC conditions, while the reduction in lead leachability exhibited a similar trend for both curing conditions. In previous studies investigating the S/S of lead contamination by AAMs, curing has predominantly been conducted at elevated temperatures ranging from 40 to 95 °C (Van Jaarsveld et al. 1998, Palomo and Palacios 2003, Palacios and Palomo 2004, Zhang et al. 2008, El-Eswed et al. 2015, Lee et al. 2016b, El-Eswed et al. 2017, Guo et al. 2017, Nikolić et al. 2018, Wang et al. 2018), with fewer studies focusing on curing within the ambient temperature range (20, 23, and 30 °C) (Van Jaarsveld and Van Deventer 1999, Phair et al. 2004, Nikolić et al. 2014, Nikolić et al. 2018). This preference is rooted in the belief that processes such as ion dissolution (silica and alumina ions), alkalinity increase (due to free water evaporation), geopolymerization reactions, and the formation of geopolymeric networks occur more efficiently at higher temperatures, thereby resulting in improved S/S performance (Kovalchuk et al. 2007). In several studies involving curing at ambient temperature, the reduction in lead leaching was notable, ranging from 2 to 12 ppm (Van Jaarsveld and Van Deventer 1999, Phair et al. 2004, Nikolić et al. 2014, Nikolić et al. 2018). Additionally, in the study by Nikolić et al. (2018), among all curing conditions examined (28 days at 20 °C, 1 day at 55 and 95 °C, and 4 h at 95 °C), the optimal S/S performance was achieved with 28 days of curing at 20 °C. Furthermore, based on the results of this study, prolonged curing under OC conditions had an adverse effect on the S/S process due to the formation of micro-cracks in the geopolymeric network induced by thermal stresses. Conversely, during long-term curing under AC conditions, the uniform and gradual development of the geopolymeric network under pozzolanic reactions in the presence of moisture led to a reduction in lead leaching in the 90-day sample compared to the 28-day sample. Consequently, it can be concluded that in the S/S process using AAMs, curing at ambient temperature does not necessarily yield weaker performance compared to curing at elevated temperatures. This characteristic renders the use of alkali-activated cements in S/S operations at sites with normal temperatures more feasible. However, when the primary objective of the S/S project is to achieve high mechanical strength of treated soil for construction activities, curing at higher temperatures will likely be more effective.

The results indicate that increasing the lead concentration generally led to a reduction in UCS and an increase in lead leaching in all cases, except for the positive effect observed on the UCS of group A treated samples. There exist diverse opinions regarding the influence of lead concentration on the S/S process. Still, the majority of reports suggest a detrimental impact of lead due to its disruption of the geopolymerization process and introduction of defects within the formation of the geopolymeric network. Negative effects on mechanical strength have been documented for lead concentrations ranging from 0.02% (El-Eswed et al. 2015), 0.5% (Zhang et al. 2008, Nikolić et al. 2014, Lee et al. 2016b), 1% (Nikolić et al. 2014, Lee et al. 2016b), 3% (Wang et al. 2018), 3.125 (Palomo and Palacios 2003), to 4% (Nikolić et al. 2018) in fly ash and metakaolin–based geopolymeric paste and mortar. Conversely, positive effects on compressive strength have been noted for lead concentrations of 0.1% (El-Eswed et al. 2015), 0.5%, 1%, and 1.5% (Wang et al. 2018) in the paste matrix of metakaolin and fly ash–based geopolymers. However, the reason behind the positive effect of lead on the geopolymerization network and the exact concentration at which this negative effect occurs remain unclear, necessitating further investigation in the lead concentration range of 0 to 1.5%. Based on the reviewed studies, it appears that all observed positive effects of lead are attributed to its presence in geopolymeric paste samples, while negative effects are more pronounced in mortar samples. In the present study, where the soil matrix was examined, the negative effects were more pronounced. The transition from cement paste to mortar and then to a soil matrix in the S/S process, accompanied by increased porosity and reduced integrity of the matrix, seems to heighten vulnerability to lead cations. This vulnerability arises from the detrimental effect of lead due to the involvement of contaminated soil particles in geopolymerization reactions and the disruption of geopolymeric network development, as well as from the aggregation of clay particles due to lead presence and increased porosity in the soil structure, ultimately leading to a decrease in the strength of contaminated samples.

This research investigates the S/S of lead-contaminated soil using alkali-activated VA. VA possesses pozzolanic properties derived from its abundant silica and aluminum content and requires minimal pre-treatment before utilization as a precursor in AAMs. VA is also known for its ease of extraction, low cost, and ability to achieve high mechanical strength and durability post-alkaline activation. These attributes have propelled its utilization in AAMs as a soil stabilizer or heavy metal immobilizer agent, garnering increasing attention from researchers. The proper application of VA in these contexts holds substantial promise for advancing sustainable development, addressing global warming, and mitigating environmental crises stemming from heavy metal pollution.

## Conclusion

The following conclusions could be drawn from the outcome of this research study:

1. Volcanic ash demonstrated high effectiveness in S/S of lead-contaminated soil. According to EPA, concentrations of 500 and 2500 mg resulted in complete control of lead leaching, while the sample with 10,000 mg/kg showed a reduction in leaching by at least 84%.
2. In all VA-treated soils, in OC condition, with different curing times, increasing the percentage of VA would increase the strength of the soil. The highest UCS was obtained for the sample prepared with 15% VA.
3. The treated OC samples exhibited significantly higher UCS compared to the AC samples. The OC condition enhances the dissolution rate of silicon and aluminum ions from the VA structure and increases alkalinity due to water evaporation. This leads to an accelerated rate of geopolymerization and the formation of a more extensive geopolymeric network, ultimately resulting in a higher UCS.
4. In OC VA-treated samples, the contaminated and uncontaminated samples reached their maximum UCS after 28 and 7 days of curing, respectively. This difference can be attributed to two factors. Firstly, the presence of Pb leads to a reduction in medium alkalinity. Secondly, the filling of soil voids by nitrate decreases the rate of water evaporation. These two factors collectively result in a reduced rate of geopolymerization and hinder the development of the geopolymeric network.
5. In the samples containing 500 mg/kg of lead and treated with volcanic ash for 1, 7, and 28 days of curing, an improvement in UCS was observed. However, as lead concentration increased to 2500 and 10,000 mg/kg, the UCS decreased at all curing times. This can be attributed to lead cations disrupting the formation of the N-(A)-S–H gel and the polycondensation process.
6. Despite the superior performance of OC curing conditions in terms of UCS results, both AC and OC conditions exhibited nearly identical outcomes in the TCLP test. This suggests that the formation of the geopolymeric network under AC conditions was adequate to prevent lead leaching, and the excess geopolymeric network formed under OC conditions had minimal impact on further controlling lead leaching.
7. Both physical encapsulation and chemical bonding mechanisms were effective in S/S of lead-contaminated soil with alkali-activated VA. However, based on the results, the chemical bonding mechanism appears to be more dominant than physical encapsulation.
8. The results of the XRD, FESEM-EDX-mapping, and FTIR tests confirmed the formation of structures such as N-A-S–H, Pb_3_SiO_5_, and hydroxy sodalite crystals, which played a significant role in controlling lead leaching.

## Data Availability

Data is available from the corresponding author with a formal request.

## References

[CR1] Álvarez-Ayuso E, Querol X, Plana F, Alastuey A, Moreno N, Izquierdo M, Font O, Moreno T, Diez S, Vázquez E (2008). Environmental, physical and structural characterisation of geopolymer matrixes synthesised from coal (co-) combustion fly ashes. J Hazard Mater.

[CR2] ASTM (1972) Standard method of test for liquid limit of soils. ASTM D423-66: 1972. American Society for Testing and Materials, West Conshohocken

[CR3] ASTM (1982) Standard method of test for plastic limit. ASTM D424-54: 1982. American Society for Testing and Materials, West Conshohocken

[CR4] ASTM (2002) Standard test method for particle-size analysis of soils. ASTM D422–63: 2002. American Society for Testing and Materials, West Conshohocken

[CR5] ASTM (2009) Standard test methods for laboratory compaction characteristics of soil using modified effort. ASTM D1557-09: 2009. American Society for Testing and Materials, West Conshohocken

[CR6] ASTM (2011) Standard practice for classification of soils for engineering purposes (Unified Soil Classification System). ASTM 2487-11: 2011. American Society for Testing and Materials, West Conshohocken

[CR7] ASTM (2016) Standard test method for unconfined compressive strength of cohesive soil. ASTM D2166-16: 2016. American Society for Testing and Materials, West Conshohocken

[CR8] ASTM (2017) Standard test method for particle-size distribution (Gradation) of fine-grained soils using the sedimentation (Hydrometer) analysis. ASTM D7928-17: 2017. American Society for Testing and Materials, West Conshohocken

[CR9] Bilondi MP, Toufigh MM, Toufigh V (2018). Experimental investigation of using a recycled glass powder-based geopolymer to improve the mechanical behavior of clay soils. Constr Build Mater.

[CR10] Bobirică C, Shim J-H, Pyeon J-H, Park J-Y (2015). Influence of waste glass on the microstructure and strength of inorganic polymers. Ceram Int.

[CR11] Bosoaga A, Masek O, Oakey JE (2009). CO2 capture technologies for cement industry. Energy Procedia.

[CR12] Chen Z, Li J-S, Zhan B-J, Sharma U, Poon CS (2018). Compressive strength and microstructural properties of dry-mixed geopolymer pastes synthesized from GGBS and sewage sludge ash. Constr Build Mater.

[CR13] Chen Y, Chen F, Zhou F, Lu M, Hou H, Li J, Liu D, Wang T (2022). Early solidification/stabilization mechanism of heavy metals (Pb, Cr and Zn) in Shell coal gasification fly ash based geopolymer. Sci Total Environ.

[CR14] Chen L, Nakamura K, Hama T (2023). Review on stabilization/solidification methods and mechanism of heavy metals based on OPC-based binders. J Environ Manag.

[CR15] Criado M, Jiménez AF, Palomo A (2010). Effect of sodium sulfate on the alkali activation of fly ash. Cement Concr Compos.

[CR16] Davidovits J (2008) Geopolymer-chemistry and application. Saint-Quentin: Institut Géopolymère 2008:585

[CR17] Deng Y-J, Yue Z-X, Wang Z-J, Huang Q, Yang X-L (2024). Optimization and mechanism of the novel eco-friendly additives for solidification and stabilization of dredged sediment. Environ Sci Pollut Res.

[CR18] Djobo JNY, Elimbi A, Tchakouté HK, Kumar S (2017). Volcanic ash-based geopolymer cements/concretes: the current state of the art and perspectives. Environ Sci Pollut Res.

[CR19] Dong Y, Xiang Y, Hou H, Lu H, Lan J (2023). Remediation of Pb–Cd contaminated soil using coal bottom ash-based geopolymer and coir: soil remodeling and mechanism. J Clean Prod.

[CR20] El-Eswed B, Yousef R, Alshaaer M, Hamadneh I, Al-Gharabli S, Khalili F (2015). Stabilization/solidification of heavy metals in kaolin/zeolite based geopolymers. Int J Miner Process.

[CR21] El-Eswed BI, Aldagag OM, Khalili FI (2017). Efficiency and mechanism of stabilization/solidification of Pb (II), Cd (II), Cu (II), Th (IV) and U (VI) in metakaolin based geopolymers. Appl Clay Sci.

[CR22] Esaifan M, Warr LN, Grathoff G, Meyer T, Schafmeister M-T, Kruth A, Testrich H (2019). Synthesis of hydroxy-sodalite/cancrinite zeolites from calcite-bearing kaolin for the removal of heavy metal ions in aqueous media. Minerals.

[CR23] Fan C, Wang B, Ai H, Qi Y, Liu Z (2021). A comparative study on solidification/stabilization characteristics of coal fly ash-based geopolymer and Portland cement on heavy metals in MSWI fly ash. J Clean Prod.

[CR24] Fernández-Jiménez A, Palomo A (2005). Composition and microstructure of alkali activated fly ash binder: effect of the activator. Cem Concr Res.

[CR25] Fernández-Jiménez A, Monzó M, Vicent M, Barba A, Palomo A (2008). Alkaline activation of metakaolin–fly ash mixtures: obtain of zeoceramics and zeocements. Microporous Mesoporous Mater.

[CR26] Fletcher RA, MacKenzie KJ, Nicholson CL, Shimada S (2005). The composition range of aluminosilicate geopolymers. J Eur Ceram Soc.

[CR27] Fu Q, Bu M, Zhang Z, Xu W, Yuan Q, Niu D (2021). Hydration characteristics and microstructure of alkali-activated slag concrete: a review. Engineering.

[CR28] Fultz B, Howe J (2012) Transmission electron microscopy and diffractometry of materials (3rd ed.). Springer Berlin Heidelberg. pp 1–57

[CR29] Geetha S, Ramamurthy K (2013). Properties of geopolymerised low-calcium bottom ash aggregate cured at ambient temperature. Cement Concr Compos.

[CR30] Golbad S, Khoshnoud P, Abu-Zahra N (2017). Hydrothermal synthesis of hydroxy sodalite from fly ash for the removal of lead ions from water. Int J Environ Sci Technol.

[CR31] Granizo N, Palomo A, Fernandez-Jiménez A (2014). Effect of temperature and alkaline concentration on metakaolin leaching kinetics. Ceram Int.

[CR32] Guo B, Liu B, Volinsky AA, Fincan M, Du J, Zhang S (2017). Immobilization mechanism of Pb in fly ash-based geopolymer. Constr Build Mater.

[CR33] Hajimohammadi A, Ngo T, Mendis P (2017). How does aluminium foaming agent impact the geopolymer formation mechanism?. Cement Concr Compos.

[CR34] Hamad R, Balzter H, Kolo K (2019). Assessment of heavy metal release into the soil after mine clearing in Halgurd-Sakran National Park, Kurdistan, Iraq. Environ Sci Pollut Res.

[CR35] Ji Z, Pei Y (2019). Bibliographic and visualized analysis of geopolymer research and its application in heavy metal immobilization: a review. J Environ Manag.

[CR36] Ji Z, Pei Y (2019). Geopolymers produced from drinking water treatment residue and bottom ash for the immobilization of heavy metals. Chemosphere.

[CR37] Kabata-Pendias A (2000) Trace elements in soils and plants. (3rd ed.). CRC Press, pp 208–217

[CR38] Khan MI, Azizli K, Sufian S, Man Z (2015). Sodium silicate-free geopolymers as coating materials: effects of Na/Al and water/solid ratios on adhesion strength. Ceram Int.

[CR39] Klima K, Schollbach K, Brouwers H, Yu Q (2022). Enhancing the thermal performance of class F fly ash-based geopolymer by sodalite. Constr Build Mater.

[CR40] Kovalchuk G, Fernández-Jiménez A, Palomo A (2007). Alkali-activated fly ash: effect of thermal curing conditions on mechanical and microstructural development–Part II. Fuel.

[CR41] Król M, Minkiewicz J, Mozgawa W (2016). IR spectroscopy studies of zeolites in geopolymeric materials derived from kaolinite. J Mol Struct.

[CR42] Lee W, Van Deventer J (2002). The effect of ionic contaminants on the early-age properties of alkali-activated fly ash-based cements. Cem Concr Res.

[CR43] Lee N, Khalid HR, Lee H-K (2016). Synthesis of mesoporous geopolymers containing zeolite phases by a hydrothermal treatment. Microporous Mesoporous Mater.

[CR44] Lee S, Van Riessen A, Chon C-M, Kang N-H, Jou H-T, Kim Y-J (2016). Impact of activator type on the immobilisation of lead in fly ash-based geopolymer. J Hazard Mater.

[CR45] Leung HM, Cheung KC, Au CK, Yung KKL, Li WC (2021). An assessment of heavy metal contamination in the marine soil/sediment of Coles Bay Area, Svalbard, and Greater Bay Area, China: a baseline survey from a rapidly developing bay. Environ Sci Pollut Res.

[CR46] Li J-S, Xue Q, Wang P, Li Z-Z (2015). Effect of lead (II) on the mechanical behavior and microstructure development of a Chinese clay. Appl Clay Sci.

[CR47] Long W-J, Ye T-H, Xing F, Khayat KH (2020). Decalcification effect on stabilization/solidification performance of Pb-containing geopolymers. Cement Concr Compos.

[CR48] Luo Y, Li S, Klima K, Brouwers H, Yu Q (2022). Degradation mechanism of hybrid fly ash/slag based geopolymers exposed to elevated temperatures. Cem Concr Res.

[CR49] Lv Y, Ma B, Liu Y, Wang C, Chen Y (2022). Adsorption behavior and mechanism of mixed heavy metal ions by zeolite adsorbent prepared from lithium leach residue. Microporous Mesoporous Mater.

[CR50] Mijarsh M, Johari MM, Ahmad Z (2014). Synthesis of geopolymer from large amounts of treated palm oil fuel ash: application of the Taguchi method in investigating the main parameters affecting compressive strength. Constr Build Mater.

[CR51] Moon J, Bae S, Celik K, Yoon S, Kim K-H, Kim KS, Monteiro PJ (2014). Characterization of natural pozzolan-based geopolymeric binders. Cement Concr Compos.

[CR52] Muhammad F, Huang X, Li S, Xia M, Zhang M, Liu Q, Hassan MAS, Jiao B, Yu L, Li D (2018). Strength evaluation by using polycarboxylate superplasticizer and solidification efficiency of Cr6+, Pb2+ and Cd2+ in composite based geopolymer. J Clean Prod.

[CR53] Nachshon U, Weisbrod N, Dragila MI, Grader A (2011) Combined evaporation and salt precipitation in homogeneous and heterogeneous porous media. Water Resour Res 47(3):W03513

[CR54] Nazari Heris M, Aghajani S, Hajialilue-Bonab M, Vafaei Molamahmood H (2020). Effects of lead and gasoline contamination on geotechnical properties of clayey soils. Soil Sediment Contam: An Int J.

[CR55] Nikolić V, Komljenović M, Marjanović N, Baščarević Z, Petrović R (2014). Lead immobilization by geopolymers based on mechanically activated fly ash. Ceram Int.

[CR56] Nikolić V, Komljenović M, Džunuzović N, Miladinović Z (2018). The influence of Pb addition on the properties of fly ash-based geopolymers. J Hazard Mater.

[CR57] Noushini A, Castel A (2016). The effect of heat-curing on transport properties of low-calcium fly ash-based geopolymer concrete. Constr Build Mater.

[CR58] Ogundiran M, Nugteren H, Witkamp G (2013). Immobilisation of lead smelting slag within spent aluminate—fly ash based geopolymers. J Hazard Mater.

[CR59] Ozer I, Soyer-Uzun S (2015). Relations between the structural characteristics and compressive strength in metakaolin based geopolymers with different molar Si/Al ratios. Ceram Int.

[CR60] Pacheco-Torgal F, Labrincha J, Leonelli C, Palomo A, Chindaprasit P (2014) Handbook of alkali-activated cements, mortars and concretes (1st ed.). Elsevier, pp 1–7

[CR61] Palacios M, Palomo A (2004). Alkali-activated fly ash matrices for lead immobilisation: a comparison of different leaching tests. Adv Cem Res.

[CR62] Palomo A, Palacios M (2003). Alkali-activated cementitious materials: alternative matrices for the immobilisation of hazardous wastes: part II. Stabilisation of chromium and lead. Cem Concr Res.

[CR63] Palomo A, Blanco-Varela MT, Granizo M, Puertas F, Vazquez T, Grutzeck M (1999). Chemical stability of cementitious materials based on metakaolin. Cem Concr Res.

[CR64] Park S, Pour-Ghaz M (2018). What is the role of water in the geopolymerization of metakaolin?. Constr Build Mater.

[CR65] Perera DS, Aly Z, Vance ER, Mizumo M (2005). Immobilization of Pb in a geopolymer matrix. J Am Ceram Soc.

[CR66] Perera D, Vance E, Finnie K, Blackford M, Hanna J, Cassidy D (2006). Disposition of water in metakaolinite based geopolymers. Adv Ceram Matrix Compos XI.

[CR67] Phair J, Van Deventer J, Smith J (2004). Effect of Al source and alkali activation on Pb and Cu immobilisation in fly-ash based “geopolymers”. Appl Geochem.

[CR68] Portland Cement Association (1971) Soil-cement laboratory handbook. Portland Cement Association, Skokie, p 59

[CR69] Pouhet R, Cyr M, Bucher R (2019). Influence of the initial water content in flash calcined metakaolin-based geopolymer. Constr Build Mater.

[CR70] Pu S, Zhu Z, Song W, Wang H, Huo W, Zhang J (2021). A novel acidic phosphoric-based geopolymer binder for lead solidification/stabilization. J Hazard Mater.

[CR71] Rees CA, Provis JL, Lukey GC, van Deventer JS (2007). Attenuated total reflectance Fourier transform infrared analysis of fly ash geopolymer gel aging. Langmuir.

[CR72] Rees CA, Provis JL, Lukey GC, Van Deventer JS (2007). In situ ATR-FTIR study of the early stages of fly ash geopolymer gel formation. Langmuir.

[CR73] Robayo-Salazar RA, de Gutiérrez RM (2018). Natural volcanic pozzolans as an available raw material for alkali-activated materials in the foreseeable future: a review. Constr Build Mater.

[CR74] Rożek P, Król M, Mozgawa W (2019). Geopolymer-zeolite composites: a review. J Clean Prod.

[CR75] Shilar FA, Ganachari SV, Patil VB, Khan TY, Dawood S (2022) Molarity activity effect on mechanical and microstructure properties of geopolymer concrete: a review. Case Stud Constr Mater 16:e01014

[CR76] Sukmak P, Horpibulsuk S, Shen S-L (2013). Strength development in clay–fly ash geopolymer. Constr Build Mater.

[CR77] Takeda H, Hashimoto S, Kanie H, Honda S, Iwamoto Y (2014). Fabrication and characterization of hardened bodies from Japanese volcanic ash using geopolymerization. Ceram Int.

[CR78] Tan L, Yu C, Wang M, Zhang S, Sun J, Dong S, Sun J (2019). Synergistic effect of adsorption and photocatalysis of 3D g-C3N4-agar hybrid aerogels. Appl Surf Sci.

[CR79] Tang L, Deng S, Tan D, Long J, Lei M (2019). Heavy metal distribution, translocation, and human health risk assessment in the soil-rice system around Dongting Lake area, China. Environ Sci Pollut Res.

[CR80] ul Haq E, Padmanabhan SK, Licciulli A (2014). Synthesis and characteristics of fly ash and bottom ash based geopolymers–a comparative study. Ceram Int.

[CR81] USEPA (1992) EPA method 1311. TCLP-toxicity characteristic leaching procedure. Publication SW-846: Test Methods for Evaluating Solid Waste, Physical/Chemical Methods. Environmental Protection Agency Washington

[CR82] Van Jaarsveld J, Van Deventer J (1999). The effect of metal contaminants on the formation and properties of waste-based geopolymers. Cem Concr Res.

[CR83] Van Jaarsveld J, Van Deventer J, Lorenzen L (1998). Factors affecting the immobilization of metals in geopolymerized flyash. Metall Mater Trans B.

[CR84] Wang Y, Han F, Mu J (2018). Solidification/stabilization mechanism of Pb (II), Cd (II), Mn (II) and Cr (III) in fly ash based geopolymers. Constr Build Mater.

[CR85] Wang S, Liu B, Zhang Q, Wen Q, Lu X, Xiao K, Ekberg C, Zhang S (2023) Application of geopolymers for treatment of industrial solid waste containing heavy metals: state-of-the-art review. J Clean Prod 390:136053

[CR86] Xia M, Muhammad F, Zeng L, Li S, Huang X, Jiao B, Shiau Y, Li D (2019). Solidification/stabilization of lead-zinc smelting slag in composite based geopolymer. J Clean Prod.

[CR87] Yaseri S, Verki VM, Mahdikhani M (2019). Utilization of high volume cement kiln dust and rice husk ash in the production of sustainable geopolymer. J Clean Prod.

[CR88] Yunsheng Z, Wei S, Qianli C, Lin C (2007). Synthesis and heavy metal immobilization behaviors of slag based geopolymer. J Hazard Mater.

[CR89] Zhang J, Provis JL, Feng D, van Deventer JS (2008). Geopolymers for immobilization of Cr6+, Cd2+, and Pb2+. J Hazard Mater.

[CR90] Zhou S, Yang Z, Zhang R, Li F (2021). Preparation, characterization and rheological analysis of eco-friendly road geopolymer grouting materials based on volcanic ash and metakaolin. J Clean Prod.

[CR91] Zhuo H, Fu S, Liu H, Song H, Ren L (2019). Soil heavy metal contamination and health risk assessment associated with development zones in Shandong, China. Environ Sci Pollut Res.

[CR92] Zuhua Z, Xiao Y, Huajun Z, Yue C (2009). Role of water in the synthesis of calcined kaolin-based geopolymer. Appl Clay Sci.

